# Supplementary material for: Accuracy of Therapeutic Drug Monitoring of Teicoplanin at the Onset of Febrile Neutropenia
Source: Medicina (Kaunas). 2023 Apr 13;59(4):758. doi: 10.3390/medicina59040758 (PMC10145105; doi:10.3390/medicina59040758)
Supplement: Supplementary file 1 [file medicina-59-00758-s001.zip › medicina-2341110-supplementary.pdf]

Table S1. Percentage of predicted TEIC blood concentration within ±25% or ±50% of measured TEIC blood concentration.

Characteristics		parameter 1		parameter 2		parameter 3	
		Percentage within ±25% of $C_{measured}$	Percentage within ±50% of $C_{measured}$	Percentage within ±25% of $C_{measured}$	Percentage within ±50% of $C_{measured}$	Percentage within ±25% of $C_{measured}$	Percentage within ±50% of $C_{measured}$
Total (n = 39)		61.5	97.4	71.8	94.9	64.1	97.4
Sex	Male (n = 28)	57.1	96.4	67.9	92.9	60.7	96.4
	Female (n = 11)	72.7	100	81.8	100	72.7	100
Age (years)	< 65 (n = 9)	44.4	88.9	66.7	88.9	55.6	88.9
	≥ 65 (n = 30)	66.7	100	73.3	96.7	66.7	100
BW (kg)	< 50 (n = 12)	83.3	100	91.7	100	66.7	100
	≥ 50 (n = 27)	51.9	96.3	63.0	92.6	63.0	96.3
BMI (kg/m^2)	< 18.5 (n = 7)	85.7	100	85.7	100	85.7	100
	≥ 18.5 (n = 32)	56.3	96.9	68.8	93.8	59.4	96.9
Alb (g/dL)	< 2.5 (n = 13)	69.2	92.3	76.9	92.3	61.5	92.3
	≥ 2.5 (n = 26)	57.7	100	69.2	96.2	65.4	100
Scr (mg/dL)	< 0.6 (n = 8)	37.5	100	62.5	100	25.0	100
	≥ 0.6 (n = 31)	67.7	96.8	74.2	93.5	74.2	96.8
Ccr (mL/min)	< 50 (n = 8)	87.5	100	87.5	100	87.5	100
	≥ 50 (n = 31)	54.8	96.8	67.7	93.5	58.1	96.8
Neutrophil count (/μL)	< 100 (n = 30)	53.3	96.7	66.7	93.3	60.0	96.7
	100–500 (n = 5)	80.0	100	80.0	100	80.0	100
	≥ 500 (n = 4)	100	100	100	100	75	100

Abbreviations: BW, Body weight; BMI, Body Mass Index; $C_{measured}$, measured TEIC blood concentration; TP, total protein; Alb, albumin; AST, aspartate transaminase; ALT, alanine transaminase; Scr, serum creatinine; BUN, blood urea nitrogen; Ccr, creatinine clearance.
